# Zika puzzle in Brazil: peculiar conditions of viral introduction and dissemination - A Review

**DOI:** 10.1590/0074-02760160510

**Published:** 2017-04-06

**Authors:** Cristina Possas, Patricia Brasil, Mauro CA Marzochi, Amilcar Tanuri, Reinaldo M Martins, Ernesto TA Marques, Myrna C Bonaldo, Antonio GP Ferreira, Ricardo Lourenço-de-Oliveira, Rita Maria R Nogueira, Patricia C Sequeira, Keyla BF Marzochi, Akira Homma

**Affiliations:** 1 Fundação Oswaldo CruzFundação Oswaldo CruzRio de JaneiroRJBrasilFundação Oswaldo Cruz-Fiocruz, Bio-Manguinhos, Assessoria Científica Sênior, Rio de Janeiro, RJ, Brasil; 2 Fundação Oswaldo CruzFundação Oswaldo CruzInstituto Nacional de Infectologia Evandro ChagasLaboratório de Pesquisa Clínica em Doenças Febris AgudasRio de JaneiroRJBrasilFundação Oswaldo Cruz-Fiocruz, Instituto Nacional de Infectologia Evandro Chagas, Laboratório de Pesquisa Clínica em Doenças Febris Agudas, Rio de Janeiro, RJ, Brasil; 3 Fundação Oswaldo CruzFundação Oswaldo CruzInstituto Nacional de Infectologia Evandro ChagasLaboratório de Pesquisa Clínica e Vigilância em LeishmaniosesRio de JaneiroRJBrasilFundação Oswaldo Cruz-Fiocruz, Instituto Nacional de Infectologia Evandro Chagas, Laboratório de Pesquisa Clínica e Vigilância em Leishmanioses, Rio de Janeiro, RJ, Brasil; 4 Universidade Federal do Rio de JaneiroUniversidade Federal do Rio de JaneiroInstituto de BiologiaDepartamento de GenéticaRio de JaneiroRJBrasilUniversidade Federal do Rio de Janeiro, Instituto de Biologia, Departamento de Genética, Rio de Janeiro, RJ, Brasil; 5 Fundação Oswaldo CruzFundação Oswaldo CruzCentro de Pesquisas Aggeu MagalhãesDepartamento de VirologiaRecifePEBrasilFundação Oswaldo Cruz-Fiocruz, Centro de Pesquisas Aggeu Magalhães, Departamento de Virologia, Recife, PE, Brasil; 6 University of PittsburghCenter for Vaccine ResearchPittsburghPAUnited StatesUniversity of Pittsburgh, Center for Vaccine Research, Pittsburgh, PA, United States; 7 Fundação Oswaldo CruzFundação Oswaldo CruzInstituto Oswaldo CruzLaboratório de Biologia Molecular de FlavivírusRio de JaneiroRJBrasilFundação Oswaldo Cruz-Fiocruz, Instituto Oswaldo Cruz, Laboratório de Biologia Molecular de Flavivírus, Rio de Janeiro, RJ, Brasil; 8 Fundação Oswaldo CruzFundação Oswaldo CruzDepartamento de Reativos para DiagnósticoRio de JaneiroRJBrasilFundação Oswaldo Cruz-Fiocruz, Bio-Manguinhos, Departamento de Reativos para Diagnóstico, Rio de Janeiro, RJ, Brasil; 9 Fundação Oswaldo CruzFundação Oswaldo CruzInstituto Oswaldo CruzLaboratório de Mosquitos Transmissores de HematozoáriosRio de JaneiroRJBrasilFundação Oswaldo Cruz-Fiocruz, Instituto Oswaldo Cruz, Laboratório de Mosquitos Transmissores de Hematozoários, Rio de Janeiro, RJ, Brasil; 10 Fundação Oswaldo CruzFundação Oswaldo CruzInstituto Oswaldo CruzLaboratório de FlavivírusRio de JaneiroRJBrasilFundação Oswaldo Cruz-Fiocruz, Instituto Oswaldo Cruz, Laboratório de Flavivírus, Rio de Janeiro, RJ, Brasil

**Keywords:** Zika, microcephaly, congenital Zika syndrome, neurological disorders, pathogenesis, epidemiology

## Abstract

This article discusses the peculiar conditions that favoured the unexpected introduction of Zika virus into the poorest northeastern region of Brazil in 2015, its speed of transmission to other Brazilian states, other Latin American countries and other regions, and the severity of related neurological disorders in newborns and adults. Contrasting with evidence that Zika had so far caused only mild cases in humans in the last six decades, the epidemiological scenario of this outbreak in Brazil indicates dramatic health effects: in 2015, an increase of 20-fold in notified cases of microcephaly and/or central nervous system (CNS) alterations suggestive of Zika congenital infection, followed by an exponential increase in 2016, with 2366 cumulative cases confirmed in the country by the end of December 2016. A significant increase in Guillain-Barré syndrome in adults has also been reported. Factors involved in viral dissemination, neural pathogenesis and routes of transmission in Brazil are examined, such as the role of social and environmental factors and the controversies involved in the hypothesis of antibody-dependent enhancement, to explain the incidence of congenital Zika syndrome in Brazil. Responses to the Zika outbreak and the development of new products are also discussed.

The emergence of Zika virus (ZIKV) (an arbovirus of the *Flavivirus* genus) in Northeast Brazil in 2015, its unprecedented speed of transmission to other Brazilian states and other Latin American countries in nearly nine months and its global spread caught the scientific community, international organisations and policy makers by surprise, with 56 countries now with reported outbreaks (WHO 2016) The exponential increase and extreme severity of ZIKV neurological disorders, congenital Zika syndrome in newborns and Guillain-Barré syndrome in adults, contrasting with evidence since its first isolation in humans in 1954 in a young girl in Eastern Nigeria (MacNamara 1954) of mild disease and non-life threatening symptoms, turned this new disease into one of the highest priorities of global public health concern.

Recent scientific findings, discussed here, have contributed to increase this global concern. In addition to the vectorial transmission by *Aedes aegypti* in many countries, as a primary Zika vector, there is now evidence indicating that there is a significant potential for global amplification of Zika outbreaks through sexual transmission, including prolonged presence of the virus in the semen, and through human blood-borne transmission, with 12 countries in different regions reporting current evidence of person-to-person Zika transmission (WHO 2016).

In contrast with this new global scenario for outbreaks, the peculiar conditions of Zika emergence in Brazil as a new disease remain poorly understood. The main challenge in addressing the complexity of Zika is then to identify the current gaps in knowledge and to conceive comprehensive conceptual and response frameworks, from interdisciplinary and multisectoral approaches, allowing us to anticipate, monitor and respond in a timely manner to outbreaks.

From this perspective, we present in this literature review the contributions from scientists from a broad range of disciplines, attempting to identify the biological, ecological and social processes involved in the peculiar conditions of Zika introduction, emergence and spread in Brazil.

*Methodological procedures: search and selection criteria* - We searched all international and national publications through December 2016, including peer-reviewed journal articles; Brazilian government bulletins and reports; international public health agency publications and reports, including the World Health Organization (WHO), Pan American Health Organization (PAHO), US Centers for Disease Control and Prevention (CDC) and European Centre for Disease Prevention and Control (ECDC). The search was conducted in PubMed, Web of Science, Scopus, ProMed and Google Scholar, using the keywords ‘Zika’ ‘ZIKV’, ‘ZIKAV’ and ‘Zika virus’. Publications were selected in accordance with their pertinence, quality and contributions to the main topics in this review.

*Background: a new disease* - On October 22nd, 2015, the state of Pernambuco in Northeast Brazil notified the Ministry of Health regarding 26 cases of microcephaly, considering them an event of importance to the public health of the state. On October 23rd, 2015, the Brazilian Ministry of Health reported the event to the WHO, in accordance with the International Health Regulations. At that time, based on preliminary epidemiological data and laboratory tests performed at FIOCRUZ-Pernambuco, mainly the identification of the virus in the central nervous fluid (CSF), the main hypothesis proposed by researchers from FIOCRUZ-Pernambuco and the Microcephaly Emergency Response Group (MERG) for the sharp increase in microcephaly cases was infection by ZIKV in pregnancy. On November 11th, 2015, based on preliminary results of clinical, epidemiological and laboratorial investigations, the Ministry of Health recognised the association between the Zika epidemic and the increased numbers of microcephaly in Northeast Brazil, which was then reclassified as a potential Public Health Emergency of International Concern. A protocol for detecting and investigating cases of microcephaly was soon established at the Ministry of Health. The WHO declared a Public Health Emergency of International Concern on February 1st, 2016, which was lifted on November 18, 2016, as identified by the WHO as an ongoing threat (WHO 2016).

Soon, it became clear that severe microcephaly is one of the symptoms in the *spectrum* of congenital Zika syndrome. The evidence in Brazil suggests that this syndrome can have a wide range of presentations and that the Zika cases with microcephaly are far more severe than the other congenital diseases resulting from exposure to other infectious agents (Freire et al. 2015, Brasil et al. 2016a, Faria et al. 2016, Rasmussen et al. 2016). In many cases, the damage to the brain of newborns, such as parts of the brain that were not formed, calcifications and other neurological and ocular pathologies, were so severe that they led to a condition nearly equivalent to anencephaly.

In addition, increases in demyelinating diseases such as Guillain-Barré Syndrome (Brasil et al. 2016c) and neuroinvasive infections (Soares et al. 2016) and six fatal cases not in foetuses or newborns have been reported (PAHO/WHO 2016a). Phylogenetic and molecular clock analyses have estimated that the ZIKV was introduced into Brazil in May 2013 (Faria et al. 2016).

The initial perplexity regarding the severe neurological disorders in the poorest northeastern region of Brazil has been followed by reports of imported congenital Zika microcephaly cases in newborns in Slovenia and Russia and autochthonous sexual transmission in adults in the US, France and Spain. The CDC reported possible ZIKV infections in 279 women in the US and its territories (Simeone et al. 2016), and the first active Zika transmission in the US has been identified by the CDC in Florida, although only one neighbourhood reported endemic ZIKV.

*Zika epidemiology: an overview* - In 2015, Zika microcephaly cases and/or alterations in the central nervous system (CNS) in newborns in Brazil increased 20-fold. In 2016, an exponential rise in the number of cases was equally reported (MS 2016a, PAHO/WHO 2016a). From an annual average of 200 confirmed microcephaly cases before the first reported case in May 2015, by the end of December 2016, 2,366 cumulative cases had been confirmed in the country. CNS alterations with evidence of congenital infection by ZIKV were included, and thus, they are now referred to as congenital syndrome associated with ZIKV infection (CSZV). Microcephaly causally related to ZIKV was confirmed if the case met criteria established by a case definition (MS 2016a, PAHO/WHO 2016b). Cases were dismissed in accordance with the following criteria: normal exams; microcephaly and/or congenital malformations confirmed to be of non-infectious causes or cases not meeting the criteria of the case definition.

Table I provides an updated overview of these cumulative CSZV in the country, with 10,867 cases reported from 8 November to 31 December 2016 (epidemiological weeks 45/2015 to 52/2016), 2,366 cases confirmed, 49 investigated/probable, 3,183 under investigation and 5,269 dismissed (MS 2017a).

**TABLE I:** Distribution of cumulative notified cases of Zika microcephaly and/or central nervous system (CNS) alterations as defined by the Surveillance Protocol. Brazil, 08 November 2015 - 31 December 2016. EW 45/2015 - EW52/2016

Brazil/Regions	Total notified 2015 - 2016	Confirmed	Investigated probable	Under investigation	Dismissed
Brazil	10,867	2,366	49	3,183	5,269
Northeast	7,023	1,804	5	1,580	3,634
Southeast	2,324	298	44	1,100	882
North	550	92	0	239	219
Central-West	716	145	0	220	351
South	254	27	0	44	183

Source of data: MS (2017a).

Fig. 1 presents the spatial distribution of CSZV cases in the country and indicates an extreme concentration of confirmed and notified cases in the Northeast region (MS 2016a). Nevertheless, as indicated in Fig. 2, CSZV notified cases were concentrated in 58% of municipalities in this region, and confirmed cases were in only 31.9% of them (MS 2016b).

**Fig. 1:**
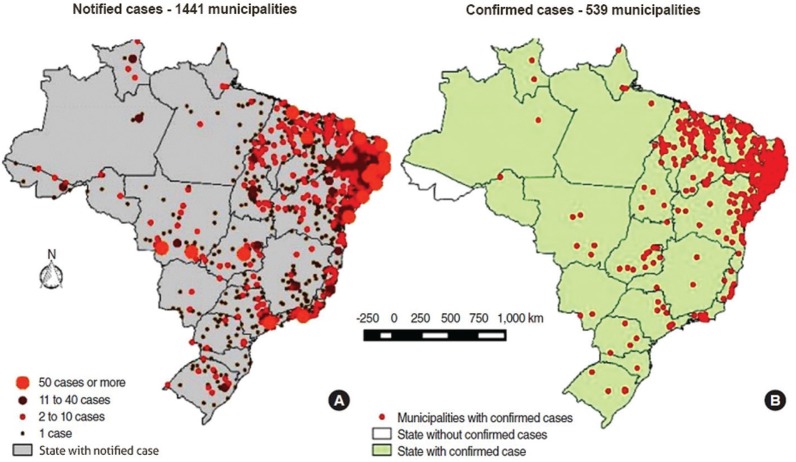
: spatial distribution of notified cases of microcephaly and/or central nervous system (CNS) alterations. Cumulative cases up to epidemiological week 21. Brazil 2016. Source: MS (2016a). Data from States and Municipalities updated up to May 28, 2016.

**Fig. 2:**
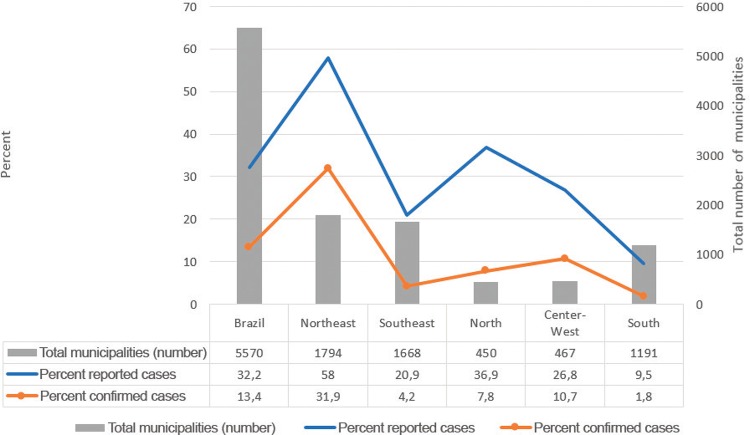
: municipalities with reported and confirmed cumulative cases of congenital Zika syndrome, in percentage of municipalities. Epidemiological week 50. Brazil, December 17, 2016. Source of data: Ministry of Health of Brazil, Epidemiological Report for epidemiological week 50/2016. Data from State and Municipalities Health Departments and Federal District (data updated up to 17/12/2016).

The geographical distribution of the incidence of Zika fever (indicated in Table II) should also be noted, despite limitations in incidence estimates in the country. Among the leading states in each region, three of them have the highest incidence: Mato Grosso, in Central-West Brazil, with 652.9 cases/100 thousand; Bahia, in Northeast Brazil, with 328.2 cases/100 thousand; and Rio de Janeiro, in Southeast Brazil, with 363.6 cases/100 thousand (MS 2017b).

**TABLE II:** Zika fever incidence per 100,000 population compared with those of dengue and chikungunya fevers in regions and states with leading incidence (in parenthesis). Brazil, 2016, up to Epidemiological Week 51 2016

Region	Chikungunya	Dengue	Zika
North (Tocantins)	44.0	222.6	73.8 (150.6)
Northeast (Bahia)	407.7	573.4	134.2 (338.5)
Southeast (Rio de Janeiro)	27.7	999.5	105.6 (407.7)
South (Paraná)	6.0	250.4	3.3 (6.1)
Central-West (Mato Grosso)	11.2	1,318.8	219.2 (670.5)

Source of data: MS (2016b).

CSZV follows the geographical spread of the Zika infection, and it should be considered that there is a time lag between infection during the first trimester of pregnancy and the detection of microcephaly. Similarly, as indicated in Table III, Colombia and other American countries, which initially had Zika infections but not CSZV cases, are now reporting them in increasing numbers (PAHO/WHO 2016b).

**TABLE III:** Countries and territories in the Americas with reported microcephaly and/or central nervous system (CNS) malformation cases potentially associated with Zika virus infection, February 2nd, 2017

Country	Number of confirmed cases
Brazil	2,316
Colombia	86
United States	42
Dominican Republic	22
Martinique	19
French Guiana	16
Guatemala	15
Other countries	87

Total	2,603

Source of data: PAHO (2017). Data provided by the health authorities of countries and territories to PAHO/WHO.

Finally, it is important to highlight, as indicated in Fig. 3, that a sharp decline has been observed in the evolution of notified cases of microcephaly and/or alterations in CNS attributed to Zika in Brazil per month of notification and per regions from 2015 and 2016 (MS 2017c). This decline, particularly sharp in the Northeastern region, might be seasonal, related to decreased mosquito infestation in winter/autumn and/or the result of postponed pregnancies caused by the increased awareness of women regarding Zika risks, which would lead, if confirmed, to a significant reduction in birth rates. This possibility of a Zika-related reduction in fertility is a concern for Brazil, a country that already has a below-replacement fertility rate of 1.8 children per woman. Despite this sharp decline in the number of ZIKV cases, they remain high and could resurface in 2017, with increased mosquito infestation in the summer, although it is expected that they might remain, for the mentioned reasons, lower than those of the outbreaks of 2015 and 2016.

**Fig. 3:**
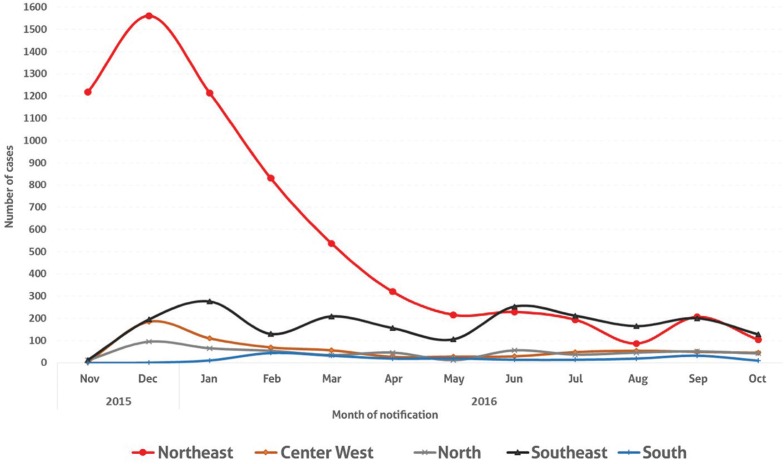
: evolution of notified cases of microcephaly and/or alterations in central nervous system (CNS) attributed to Zika per month of notification and regions. Brasil, 2015 and 2016. Source: MS (2017c). Data provided by Health Departments of States and Municipalities up to 17/12/2016.

*Routes of transmission: complexity and uncertainty* - The Zika outbreak in Brazil has led to an unprecedented situation. For the first time, a mosquito-borne virus is causing, besides widespread neurological disorders, birth defects. Similar to other epidemic arboviruses, such as chikungunya, yellow fever and dengue (at least, serotype 2), ZIKV has emerged from an African sylvatic transmission cycle ensured by *Aedes* mosquitoes. These viruses co-evolved with mosquitoes essentially belonging to the subgenus *Stegomyia* of *Aedes*, of which the most spread species is *Ae. aegypti.* Therefore, any territory infested by this mosquito is receptive to the transmission of any of these viruses. Moreover, they share the vector, although the natural co-infection of mosquitoes with Zika and others of these viruses has not been reported yet.

Vector competence to ZIKV in American *Aedes* mosquito populations has been shown to be heterogeneous (Richard et al. 2016). One *Ae. aegypti* and one *Ae. albopictus* population from Brazil and USA, respectively, challenged with a ZIKV strain from the Asian lineage isolated from New Caledonia exhibited high-to-moderate infection and dissemination rates but an essentially low transmission efficiency. By contrast, a very high transmission rate was found for a Brazilian *Ae. aegypti* population challenged with a ZIKV isolate from the Northeastern region of the country (Chouin-Carneiro et al. 2016). Natural infections by ZIKV only in *Ae. aegypti* in Brazil confirmed this mosquito as the only primary vector in the country, as well as in South America (Ferreira-de-Brito et al. 2016).

The possibility of transmission by other species of mosquitos, such as *Culex*, was investigated, and some suggestive evidence was found (Ayres 2016), but its role in transmission was not validated by the findings of a study conducted at FIOCRUZ in Rio de Janeiro (Fernandes et al. 2016).

Mosquito transmission of the arboviruses ZIKV, CHIKV and Dengue in Brazil can occur during all months of the year in most states, favouring fast dissemination.

Another concern is the potential establishment of a sylvatic cycle of ZIKV in the New World. Recently, saliva samples of a few capuchins and marmosets from Northeast Brazil tested positive for ZIKV when screened by molecular methods (Favoretto et al. 2016). The possible role of New World non-human primates as ZIKV amplifiers and the vector competence to ZIKV in neotropical sylvatic mosquitoes must urgently be addressed.

Several other contributing factors should also be considered, clarifying their role in the Brazilian outbreak, such as the large naïve population for ZIKV, the high densities of competent populations of mosquito vectors and the possibility of other routes of transmission, such as sexual transmission. In the USA, the CDC confirmed cases of sexual transmission of ZIKV in American travellers returning from transmission areas and infecting sexual partners. Multiple probable cases of sexual transmission have since been reported in several countries and in Brazil (Coelho et al. 2016, D’Ortenzio et al. 2016, Deckard et al. 2016, Mansuy et al. 2016a).

Two recent breakthrough studies have identified prolonged presence of the virus for six months in the semen of two Italian men exposed to the virus in Haiti (Nicastri et al. 2016). Previously, researchers in France had found the virus in the semen three months after exposure in a patient returning from a non-epidemic area in Thailand (Mansuy et al. 2016b).

Infective particles and/or viral genomes of ZIKV have also been found in Brazil in saliva and urine (Bonaldo et al. 2016), but we do not know yet their epidemiological relevance. Research is necessary to confirm these observations and to evaluate the role of other potential routes of transmission, such as nasal fluids, the conjunctivae, as well as blood and vaginal secretions, clarifying their relative contribution to the extent of Zika dissemination.

*ZIKV: neuroinvasiveness and pathogenesis* - The full genome of ZIKV has been recently published by Brazilian researchers (Faria et al. 2016). There are two lineages of the ZIKV: the African lineage and the Asian lineage. The ZIKV outbreak in Brazil, as well as in all American countries, is due to the Asian lineage, and the isolated strains are very close to those circulating in the French Polynesia outbreak in 2013-2014 (Haddow et al. 2012, Freire et al. 2015).

Until recently, there was no evidence of viral mutations, but a recent study on the molecular evolution of the virus has identified unique nucleotide mutations in the Natal_RGN, ZKV2015, Rio-U1, and Rio-S1 strains among all 29 current human strains within the Asian lineage (Wang et al. 2016). There is so far no evidence associating viral nucleotide and amino acid (aa) changes with a more virulent phenotype. However, a recent study with human strains from the recent outbreaks revealed 34 aa modifications in comparison with the Asiatic ancestor strain P6-740 isolated from *Ae. aegypti* in Malaysia (Marchette et al. 1969). Nonetheless, there are some limitations in this comparison since the P6-740 strain has been continuously passaged in suckling mice (six times), Vero cells (one time), BHK cells (one time), and C6/36 *Ae. albopictus* cells (one time), as described in GenBank access number HQ234499. Consequently, some nucleotide and aa changes may have resulted from the passage history.

Further studies are needed to elucidate the sequential acquisition of these changes and their individual contributions to human pathogenesis and vector competence.

An important factor of genetic diversity would be genomic recombination. It was reported that during its emergence, ZIKV underwent 13 recombination events, which is considered an unusual feature for a flavivirus, which is often genetically restricted by the need to replicate in evolutionarily disparate invertebrate (mosquitoes) and vertebrate hosts (Faye et al. 2014). Notably, these observations refer to the nucleotide sequences of P6-740 (HQ234499 and KX377336), and these sequences do not contain undetermined nucleotides (N).

The pathogenesis of Zika is still unknown. The determinants and conditions leading to symptomatic or asymptomatic cases, to the severity of neurological disorders and to the patterns of disease dissemination are not yet clear (Possas 2016). Virological, clinical, immunological and histopathological studies are necessary in integrated approaches to clarify the unknown aspects involved in the aetiopathogenesis of the clinical forms of Zika.

Despite these knowledge gaps, evidence on ZIKV neurological disease, as in other flaviviruses (Muñoz et al. 2016), and on birth defects caused by its infection (de Carvalho et al. 2016, Meaney-Delman et al. 2016) is rapidly increasing. A study by Slovenian researchers provided some of the first indications of Zika neuroinvasiveness, with a case of microcephaly in the baby of a Slovenian mother who had returned from a trip to Northeast Brazil, where she had the symptoms of Zika fever and rash. The mother had a legal abortion, and after consent, the ZIKV was completely sequenced from the foetal brain (Mlakar et al. 2016). Subsequently, in Brazil, a breakthrough study identified the presence of ZIKV RNA in the amniotic fluid of two pregnant patients from Campina Grande, Paraíba (Calvet et al. 2016). This study showed for the first time in Brazil that the ZIKV can cross the placental barrier and possibly cause cases of neurological disorders in newborns.

At about the same time, a team of Brazilian researchers from Rio Grande do Norte state in Northeast Brazil and the CDC reported that the ZIKV had been found in the brains of two newborns who died soon after birth (Martines 2016). This was the first time that the direct impact of Zika on the brains of babies was clearly demonstrated in Brazil. Two recent studies reinforced the causal link between Zika infection in pregnancy and microcephaly (Cordeiro et al. 2016, de Araújo et al. 2016).

A pioneer Brazilian study with a cohort of pregnant women in Rio de Janeiro provided strong evidence on the impact of ZIKV infection, indicating that it is associated with severe outcomes, including foetal death, placental insufficiency, foetal growth restriction, and CNS injury (Brasil et al. 2016b). This study indicates that although Zika microcephaly tends to be, as in rubella and other congenital infections, more associated with infections in the first trimester of pregnancy, in some cases, other neurological disorders and adverse effects result from Zika infections in the second and third trimesters, such as foetal deaths at 36 and 38 weeks of gestation, *in utero* growth restriction with or without microcephaly, ventricular calcifications or other CNS lesions and abnormal amniotic fluid volume or cerebral or umbilical artery flow. These results are also consistent with the findings of a recent CDC mathematical modelling study indicating that microcephaly tends to be more related to infections during the first trimester of pregnancy (Reefhuis et al. 2016).

Three studies in mouse models have also confirmed some of these results on the neuroinvasiveness of the ZIKV. Researchers at the University of São Paulo have reported that the Brazilian strain of the virus could cross the placentas of pregnant mice, resulting in microcephaly in developing embryos (Cugola et al. 2016). In another study, Chinese researchers from the Chinese Academy of Sciences reported similar findings after studies on a different infection route (Li et al. 2016). Finally, in a third study, researchers at Washington University in Saint Louis found that Zika can infect pregnant, immunodeficient mice and their foetuses, causing placental damage associated with restricted growth and foetal demise (Miner et al. 2016).

In April 2016, a review study led by CDC researchers concluded that sufficient evidence had accumulated to infer a causal relationship between prenatal ZIKV infection and microcephaly and other serious brain anomalies (Rasmussen et al. 2016).

*Zika and dengue interactions: the controversy* - In Brazil, the geographical overlaps of DENV and ZIKV infections in many states where severe Zika alterations had emerged after a large dengue outbreak attracted the attention of scientists and government authorities. In 2015, when severe Zika neurological alterations emerged in newborns and adults, Brazil had reported 1,649,008 cases of dengue, an increase of 178% compared to 2014 (MS 2016c). Zika neurological alterations following dengue outbreaks have also been reported in other Latin American countries and other regions of the globe.

The intense scientific debate on the immune mechanisms possibly involved in this overlap highlighted the need for a better understanding of the immune responses that might be involved in the possible interactions of both flaviviruses. The main challenge has been to clarify the possible impacts of previous immunity to DENV through infection or vaccination on protective immunity and/or disease enhancement in individuals exposed to ZIKV infection. Despite recent advances in experimental research in this area, the antibody-dependent enhancement (ADE) hypothesis remains controversial in the scientific community since many gaps in knowledge persist, particularly in clinical and epidemiological research.

On the one hand, recent breakthrough experimental studies have provided significant evidence supporting the hypothesis that the dengue virus may contribute to ADE and severe disease in Zika infections. The results of an *in vitro* study surprised researchers with high levels of Zika replication detected in the presence of dengue antibodies in cultured immune cells (Paul et al. 2016). In sequence, another study has also reported similar results, showing that certain different antibodies to dengue virus react to Zika but not strongly enough to neutralise the virus (Dejnirattisai et al. 2016). Instead, when blood plasma from patients who had recovered from dengue was added to cell cultures infected with Zika, this study found that the ZIKV replication increased by 100-fold. Other experimental studies on ADE have provided evidence in this same direction (Barba-Spaeth et al. 2016, Priyamvada et al. 2016, Rivino & Lim 2016).

On the other hand, the opponents of this ADE hypothesis argue that this enhancement would not be specific for Zika since flavivirus antibodies have been known for decades in the scientific literature to cross-react with other flavivirus antigens, including those of dengue virus, when diluted to the adequate concentration. However, this argument does not permit dismissing the possibility that ADE may contribute to the severity of Zika cases in newborns and adults.

Concerns raised by the aforementioned *in vitro* studies should not be thus ignored. The finding that dengue virus antibodies may partially neutralise ZIKV and strongly stimulate its replication *in vitro* should not be disregarded at least as a hypothesis for further investigation.

Although these results refer to *in vitro* ADE and not *in vivo* ADE, as many scientists argue, if clinically and epidemiologically confirmed, they may have detrimental implications for the development of vaccines. Dengue and Zika vaccines might induce antibody enhancement against both viruses, with severe adverse effects in vaccinees. Many gaps in knowledge persist regarding the immunity to ZIKV (Rivino & Lim 2016) and interactions with other flaviviruses, and additional research will be necessary to clarify these issues.

In addition, it should also be highlighted that many issues concerning Zika ADE related to other flavivirus outbreaks in Brazil remain unclarified. In addition to a previous large dengue outbreak, Brazil has also experienced large outbreaks of chikungunya, as indicated in Table II, raising hypotheses on possible interactions of these viruses that might explain the severe neurological disorders in newborns and adults. The recent sylvatic yellow fever outbreak in Brazil (MS 2017c), considered the largest outbreak in the country in six decades and with a potential for urbanisation, could become an additional factor in these complex interactions.

Consequently, global support of scientists is imperative for a better understanding of the immune mechanisms involved in the severe alterations of Zika congenital syndrome and their possible association with ADE. It is also necessary to investigate other unknown intervenient factors, whether genetic, immunological, virological, environmental or social, which could play a role in the severe neurological alterations of the ZIKV in Brazil (Brasil et al. 2016c, Fauci & Morens 2016, Possas 2016).

*Breakthroughs: diagnostic tools, vaccines and drugs* - New products are urgently needed to address the rapidity of Zika transmission. A recent study has determined for the first time the structure of ZIKV and specific regions in this structure, providing a fantastic visualisation of the virus at near-atomic resolution (Sirohi et al. 2016). In general, the envelope structure of the ZIKV is similar to other flaviviruses, except for the region surrounding the N-glycosylation site. These findings open possibilities in the development of Zika vaccines and therapeutic molecules.

A major issue is the scarcity of diagnostic tools for Zika. Laboratorial diagnosis of ZIKV infection currently relies mostly on acute-phase samples for the detection of viral RNA by real time reverse transcriptase polymerase chain reaction (RT-PCR) tests, with limitations due to the short period of viraemia. Viral isolation in cultured cells and the determination of plaque forming units can also be performed in acute samples, but this method is restricted to very specialised laboratories.

Specific serological tests for the detection of anti-ZIKV IgM and IgG antibodies are the most important bottleneck of ZIKV infection diagnosis so far. Due to the high prevalence of dengue seropositivity in the Brazilian population, the results obtained from serological tests cannot be trusted due to cross-reactivity, hindering a seroprevalence study of ZIKV infection.

Another crucial issue is the development of a vaccine. Vaccine development against Zika is at an early stage, as it emerged in response to recent outbreaks. There are several vaccine candidates. The US National Institutes of Health-NIH is developing a DNA-based Zika vaccine, and there are now 18 other agencies and companies around the world developing Zika vaccines. Recently, Inovio Pharmaceuticals and GeneOne Life Science from South Korea announced approval to initiate a phase I human trial to evaluate Inovio’s Zika DNA vaccine, which has induced robust antibody and T cell responses in small and large animal models. Other international initiatives funded by anonymous private donors, such as the Zika Cure Alliance (Cura Zika) by the University of Pittsburgh in cooperation with Fiocruz in Brazil, are also supporting vaccine development. These new vaccine candidates are mostly based on platforms for dengue vaccine: inactivated, live attenuated, live vectored, chimeric, virus-like particles (VLP), subunit protein, DNA. Several Brazilian institutes are now involved in this effort. Bio-Manguinhos/Fiocruz is developing Zika vaccines in different partnerships and platforms: inactivated, 17 D Yellow Fever/Zika chimeric virus in tissue culture (Bonaldo et al. 2014), subunit E protein and VLP expressed in tobacco. The Butantan Institute is developing an inactivated vaccine in partnership with the US Bio-medical Advanced Research and Development Authority (Barda). Another recent study in collaboration between Harvard University and the University of São Paulo has shown that a single immunisation of a plasmid DNA vaccine or a purified inactivated virus vaccine provides complete protection in susceptible mice against challenge with a ZIKV outbreak strain from Northeast Brazil (Larocca et al. 2016). Finally, the University of Pittsburgh in collaboration with the Fiocruz/Aggeu Magalhães Research Centre is now developing a Zika vaccine using a new version of LAMP technology under the sponsorship of the Cura Zika programme.

It is important to identify new pathways for the accelerated development of a Zika vaccine, as this is a global public health concern. Unfortunately, in Brazil, the current regulatory legislation does not provide mechanisms for speeding up clinical trial evaluations, such as “fast track”, “expedited review”, “priority review”, “accelerated approval”, or “rolling review”, and thus, progress for vaccine development may be slower than needed.

Concerning the development of antiviral treatments for Zika, it should be stressed that there are so far no antivirals for any flavivirus. New drugs are urgently needed in the Brazilian outbreak. As a recent study has indicated, although the scientific community has known about the ZIKV for 60 years and advanced molecular biology technologies are available, the lack of appropriate *in vitro* assays for Zika hampers researchers’ ability to quickly identify and test molecules that might possess antiviral activity (Ekins et al. 2016). The US National Institute of Allergy and Infectious Diseases is trying to overcome these barriers, using its existing antiviral drug screening programme for other *flaviviruses*, such as dengue, West Nile, yellow fever, and Japanese encephalitis (NIAID/NIH 2016). NIAID has evaluated 60 antiviral compounds for activity against Zika, with 15 of them found to have moderate to high activity. The goal is to develop a broad-*spectrum* antiviral drug that can be used to treat a variety of *flaviviruses*, including Zika (NIAID/NIH 2016). Other promising studies are ongoing with nucleoside inhibitors of ZIKV but still using *in vitro* assays (Eyer et al. 2016). Finally, in Brazil, a recent study has concluded for the potential of an anti-HCV drug, the clinically approved antiviral drug Sofosbuvir, for a secondary use against ZIKV (Sacramento et al. 2017).

*Final considerations* - The findings presented in this review indicate remarkable scientific advances and breakthroughs but also highlight the need to overcome the remaining gaps in knowledge, such as the infectivity of the circulating Zika viral strain from French Polynesia introduced into Northeast Brazil, pathogenesis, immunity in regions with previous outbreaks of other flaviviruses, levels of viraemia, duration and risks of viral persistence, routes of transmission and risk of sexual transmission and the complex eco-social conditions favouring its emergence and spread.

These gaps and the rapid spread of ZIKV virus worldwide allow us to anticipate an increasing global burden, particularly affecting the poorest developing countries. A broad perspective supporting collaboration and multisectoral partnerships will be crucial to clarify the complex viral, genetic, immunological, environmental and social factors involved in the rapid spread of the virus and in the exponential increase in CSZV in the country. Poverty, inadequate infra-structure, such as limited access to sanitation and garbage collection, and inadequate vector control in Brazil have contributed to a very high density of *Aedes* mosquitos and have thus created conditions favouring the emergence and rapid dissemination of the ZIKV. This implies a requirement for large-scale public health interventions. There is also an urgent need to accelerate new, innovative preventive and therapeutic strategies and responses. Scientists and technologists from Brazil and other developing countries should be supported and actively involved in this global effort.
